# Editorial: Novel Insights Into the Response of the Plant Microbiome to Abiotic Factors

**DOI:** 10.3389/fpls.2021.607874

**Published:** 2021-05-28

**Authors:** Feth el Zahar Haichar, Tomislav Cernava, Jia Liu, Collin M. Timm

**Affiliations:** ^1^INSA-Lyon, Université Claude Bernard Lyon1, CNRS, UMR5240, Microbiologie, Adaptation, Pathogénie, Univ Lyon, Villeurbanne, France; ^2^Institute of Environmental Biotechnology, Graz University of Technology, Graz, Austria; ^3^Chongqing Key Laboratory of Economic Plant Biotechnology, College of Landscape Architecture and Life Science/Institute of Special Plants, Chongqing University of Arts and Sciences, Yongchuan, China; ^4^Johns Hopkins University Applied Physics Laboratory, Laurel, MD, United States

**Keywords:** plant microbiome, plant-microbe interactions, abiotic stress, nutrient limitation, agricultural practices

The plant-associated microbiota was previously shown to play a crucial role in plant health and growth through its ability to protect plants against pathogens, increasing plant nutrient uptake, modulating plant hormone signaling, and improving abiotic stress tolerance (Berg et al., 2014; Haichar et al., 2014). Recently, it was shown that certain members of the microbiota can even holistically shape disease resistance of their host plant (Matsumoto et al., 2021). Under adverse growth conditions, the diversity and function of microorganisms within the plant's native microbiota can be affected directly by environmental stresses or indirectly *via* specific plant responses (Naylor et al., 2017; Santos-Medellín et al., 2017; Timm et al., 2018). In addition to climatic factors, agricultural management and especially agrochemical inputs were identified as major drivers of microbiome shifts (Wang and Cernava, 2020). Commonly applied agricultural practices can affect up to 50% of naturally occurring microorganisms in crop plants (Chen et al., 2020). A better understanding of microbiome responses to environmental changes and host adaption will contribute to the development of new management strategies that will improve plant stress tolerance and increase plant productivity *via* targeted microbiota modulation (Figure 1). Advances in the development of new high-throughput sequencing technologies and other methods for assessing complex microbiomes will help to achieve this challenge (Berg et al., 2020). The current *Frontiers in Plant Science* Research Topic of nine articles sheds light on how the plant microbiome responds to changes in the environment, such as drought, salt stress, climate change, environmental pollution, and agricultural plant treatments. Furthermore, the included articles contribute to the understanding of processes involved in plant holobiont assembly and provide additional clues how the environmental and human activities play a role in them.

**Figure 1:**
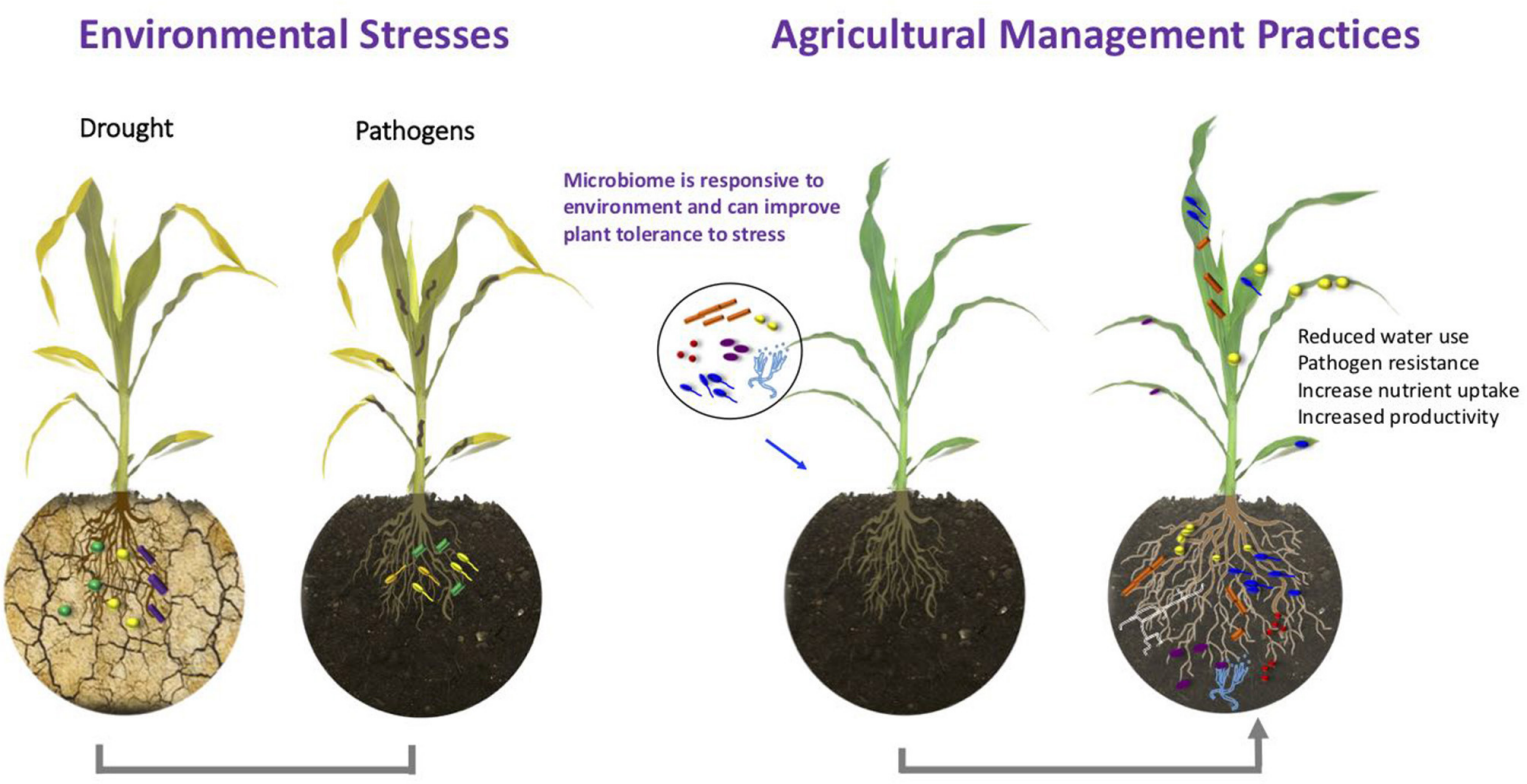
Plants can recruit and maintain microbial communities that can affect their health, fitness, and productivity when exposed to external stressors such as drought and pathogens. Inoculation of plants with microorganisms or modulation of the microbiome via various amendments can increase drought tolerance, improve nutrient uptake, and disease resistance. These practices provide valuable management strategies for sustainable agriculture.

One strategy to meet global food demands is to further boost crop yields. Crops are largely impacted by a variety of biotic and abiotic stresses, such as increasing pathogen pressure and drought. In one of the articles, the plant microbiome response to drought conditions was studied by Simmons et al.. The authors demonstrated through 16S rRNA gene fragment sequencing that the degree of drought correlates with enrichment of *Actinobacteria* in four species of millet and that this enrichment occurs along the length of the root. The authors also provided evidence that the enrichment only occurs in roots that are directly exposed to drought, therefore it is not likely evoked due signaling within the root system

Crop fitness also depends on the seed microbiota, which can be altered during storage in response to fumigation. Solanki et al. characterized the composition of the fungal and bacterial communities in stored wheat grains and determined the impact of phosphine fumigation on the wheat grain microbiome during storage. They found that phosphine fumigation had a significant impact on the diversity and abundance of various components of the wheat grain microbiome. Indeed, highly abundant bacteria, such as *Streptomyces, Prevotella, Bilophila*, as well as unidentified groups of *Parabacteroides* disappeared after phosphine treatment. This change corresponded with a significant increase in *Bacillus* representatives. Distinct species of this genus are known to be able to use phosphine and hence to take advantage of this ability to enhance their survival and proliferation. The same result was observed for fungi with, for example, a greater number of *Fusarium* species were detected after changes in microbial community composition following phosphine fumigation and correlated with the presence of *Fusarium* toxins. More research will be needed in the future to understand pesticide-evoked microbial community changes in order to develop strategies to overcome mycotoxin contaminations and other adverse effects.

To prevent food loss different post-harvest treatments are nowadays applied such as hot water treatment (HWT). Wassermann et al. have investigated the microbiome responses to HWT in apples, a sustainable method to reduce pathogen-induced postharvest fruit decay. They demonstrated by combining metabarcoding analysis and real time qPCR that only minor shifts were caused by HWT within the fungal microbiome while the bacterial community was insignificantly affected. These results suggested that HWT treatment was highly effective in reducing rot symptoms on apples while preserving the natural microbiota. Pathogen infection on the other hand, was shown to significantly affect the bacterial community. A total of 18 bacterial and 4 fungal taxa were shared between HWT-subjected and untreated, healthy apples, while being absent in diseased apples. These taxa may participate in reducing post-harvest decay and present promising candidates for biological control. Interestingly, the authors evidenced that by combining a biological control consortium (previously isolated from apples) and HWT, the total infection rates can be reduced up to 42%. In summary, this study provides various evidences that a combination of methods is most promising, in terms of sustainable approaches, to reduce post-harvest decay of apples.

Commonly applied management practices for fruit processing (organic and conventional) are connected to defined abiotic treatments pre- and post-harvest and thus are likely to impact fruit-associated bacteria. The study by Wassermann et al. conducted a study designed to identify (i) differences in microbial community composition in defined tissues of apples fruits and (ii) the impact of management practices on apple-associated bacteria. This study is of particular interest, because apples are the most consumed fruits worldwide and fruit-associated microbiota might transiently colonize our gut; thus influence our own microbiota. Using 16S rRNA gene amplicon analyses with qPCR experiments, and complementary fluorescence *in situ* hybridization combined with confocal laser scanning microscopy (FISH-CLSM) to visualize the presence of bacteria, the authors identified tissue-specific (stem, peel, fruit pulp, seeds, and calyx) and management-specific microbiome structures. Each of the analyzed apple tissues was found to be colonized by distinct bacterial communities. Fruit pulp and peel were characterized by a high diversity and low bacterial abundance. In contrast, seeds were less diverse than other tissues, but harbored the highest bacterial abundance. Complementary visualizations by FISH-CLSM indicated the highest bacterial abundance on stem, stem end, and calyx end samples, whereas peel and fruit pulp were less colonized. The management practice was found to significantly influence the microbiome of all tissues within apples. The microbiome composition was distinct between organic and conventional tissue analogs with a strong reduction in bacterial diversity and evenness in conventionally managed apples. The abundance was not affected by the management practices, suggesting that a similar quantity of bacteria occupy tissues of organically and conventionally produced apples. Notably, the authors identified signature taxa in conventional apples, among which were OTUs assigned to *Escherichia-Shigella* known to potentially affect human health. The authors hypothesize that the highly diverse microbiome of organically managed apples might limit the effects of human pathogens, through competition.

Köberl et al. used a large-scale approach to study the response of the soil microbiome in vineyards and orchards to different agricultural practices. The impact of soil parameters as well as usage of herbicides were also investigated. Comparative analyses based on microbial profiling of prokaryotic 16S rRNA gene fragments and the fungal ITS region revealed specific adaptions of the microbial community composition to agricultural treatments, especially at higher taxonomic levels. *Proteobacteria, Acidobacteria*, and *Verrucomicrobia* were the most dominant bacterial phyla in Styrian vineyards, orchards and other agriculturally used soils. They are known to harbor plant-associated microorganisms, thus their adaption to agricultural management practices likely also has consequences for their plant hosts. Within the fungal communities, *Ascomycota* represented the most dominant fungal phylum followed by *Zygomycota*, and *Basidiomycota*. Vineyards revealed a significantly higher fungal diversity in combination with a distinct fungal community composition when compared with orchards and other agricultural soils, whereas the prokaryotic diversity was unaffected by soil usage. Soil pH was identified as the most important driver of the microbial community structure among edaphic modulators in both vineyards and orchards soils. In general, the authors showed that responses to distinct soil parameters differ in orchards and vineyards as well as that bacterial and fungal community showed different responses to the same abiotic parameters. In comparison to orchards, the microbiome of vineyards soils maintained higher stability in term of taxonomy and inferred functionality when herbicides were applied. In contrast to vineyards, where the application of herbicides was associated with a general reduction in microbial soil diversity, orchards soils were characterized by drastic shifts in terms of community composition at taxonomic and predicted functional level. This study provides deepening insights on how soil management practices affect the microbiome in agricultural soils and thus an important basis for soil microbiome management in the future.

One of the major abiotic stresses which has a severe negative impact on agricultural productivity globally is salt stress. Tisarum et al. investigated the putative role of Arbuscular Mycorrhizal Fungi (AMF) in salt stress regulation of upland pigmented rice cv. Leum Pua (LP) and compared it with a salt tolerant cultivar (cv. Pokkali; PK). LP plantlets subjected to 0 or 150 mM NaCl were inoculated with *Glomus etunicatum* (GE), *Glomus geosporum* (GG), and *Glomus mosseae* (GM) strains and compared to the salt tolerant cultivar. Among the tested AMF, GE inoculation was found to induce soluble sugar (fructose) and free proline production in host rice plants that act as osmolytes to reduce salt toxicity and hence physiological processes of LP were maintained at stable levels compared to LP without AMF inoculation. Changes in morphological and phenotypic characteristics in cvs. PK and LP under control conditions and salt stress were also observed. Interestingly, in the pericarp of rice grains inoculated with AMF and subsequently exposed to salt stress, anthocyanin accumulation was clearly observed after GE inoculation. This study underscores the important role of AMF inoculation in reducing salt toxicity rice crops and provides new perspectives to employ AMF for salt stress alleviation.

Most studies on the response of microbiome to abiotic factors were conducted below-ground in the past. Less is known about endophytic communities associated with the phyllosphere, the above-ground plant tissues. Firrincieli et al. analyzed phyllosphere endophytic bacterial communities colonizing wild *Populus trichocarpa* (black cottonwood) plants growing in native, nutrient-limited environments including hot-dry (xeric) riparian zones (Yakima River, WA), riparian zones with mid hot-dry (Tieton and Teanaway Rivers, WA), and moist (mesic) climates (Snoqualmie, Skykomish and Skagit Rivers, WA). The sampling sites had experienced different historical drought regimes as a consequence of differences in temperature, vapor pressure deficit, and precipitation. Only *Enterobacteriaceae* and *Pseudomonadaceae* occurred in a high relative abundance in the core microbiome of the poplar trees. Several plant-associated representatives of these bacterial families are known to be able to secrete specific secondary metabolites or to produce enzymes that enhance drought tolerance. In addition, the authors found that the number of observed bacterial species and the overall diversity tended to be lower in the phyllosphere of plants inhabiting the hot-dry environment. They conclude that the major driver of variation was the sampling site while climate and weather data had only a limited effect.

Little is also known about the role of air-carried microbes in the context of plant microbiome assembly. Abdelfattah et al. investigated the role of the atmosphere as a source for the recruitment and dissemination of the plant microbiota. The authors hypothesized that the atmospheric microbiome influences the composition of the fungal communities associated with the aboveground organs (flowers, fruit, and leaves) of table grapes. Surprisingly, the authors found that the atmosphere surrounding the grape phyllosphere had a significantly higher level of fungal diversity than the grape phyllosphere itself. They also found that 92% of the atmospheric fungal community originated from the local plants (grapevines). In contrast, only 4–35% of the plant-associated fungi originated from the atmosphere, providing evidence that the plants were the major source of their own microbiota. These results demonstrated that the atmosphere serves as a complementary source of recruitment for fungal communities inhabiting leaves, flowers, and fruits.

The effect of soil-applied biochars derived from different sources, on soil physicochemical properties, plant performance, and rhizosphere microbiota in durum wheat was analyzed by Latini et al. Two durum wheat varieties exhibiting different behavior and traits and two biochars (from wood chips and wheat straw pellets) were grown in a greenhouse using a low- nutrient gleyic fluvisol containing a very small amount of Pb and Zn with the aim of assessing the effect of biochar type and/or durum genotype on the diversity and composition of the microbiome associated with rhizosphere soil, soil properties, and plant growth. The authors found that the soil, plants, and rhizosphere microbial communities are affected by biochar addition. This might be linkable to general properties of biochar amendments, they enhance soil carbon content, pH and cation exchange capacity. Interestingly, biochar from a straw-based feedstock was more suitable then woody biochar for improving crop yields as its porous arrangement, distribution, and size, creates a large surface area that improves the soil and plant connection, resulting in enhanced plant growth. Biochar also had a positive effect on the soil, which possessed both zinc and lead contamination. The use of a biochar amendment resulted in a decrease in the Zn available to plants, justifying its use for the remediation of soils with low levels of heavy metal contamination. By implementing high-throughput 16S rRNA gene fragment amplicon sequencing, the authors observed that biochar treatments but not the wheat genotype had a significant influence on microbial α-diversity in the rhizosphere and that nutrient-rich biochar did not negatively impact the rhizosphere bacterial species richness, but rather stabilized it. In the future, more comparative studies will be needed to compare different biochar-cultivar interactions to determine combinations that consistently enhance yield.

In summary, the contributions of this Research Topic have allowed to decipher several detrimental effects of prevalent abiotic stress factors on the plant microbiome, including management practices used to increase production and preserve products. Across these studies, the authors investigated a variety of adverse growth conditions or human inputs that have potential to affect microbiome. Of these 9 studies, natural abiotic stresses including drought and high temperature showed reduced microbial diversity in the rhizosphere or phyllosphere communities. Management practices including pesticide application and addition of untreated woody biochar led to reduced grain and soil diversity, while activated biochar retained soil microbial diversity and hot water treatment of fruits results in increased microbial diversity. More broadly, these studies showed that non-organic agricultural soils have the lowest microbial diversity compared to vineyards or orchards, and that organic production of fruits can increase diversity in the fruit tissues, potentially affecting fruit quality and longevity. They may even have implications for human health that remain to be investigated further. The overall results also suggest that maintenance of a diverse microbial community is important when considering agricultural production, as various environmental and human induced factors can lead to reduction of soil microbial diversity. Reductions in soil microbial diversity may ultimately feedback on plant health, instigating a negative feedback loop on productivity from these systems. More research to understand implications of natural and human factors on soil microbial diversity is needed and will inform long term sustainability and soil health. There still important knowledge gaps that remain to be closed in the future. Many of them are related to common agricultural practices like the application of agrochemicals; they are widespread, yet we are only beginning to understand how they interfere with plant and soil microbiomes (Wang and Cernava, 2020). The findings in this Research Topic and upcoming topics will serve as a basis to improve agricultural management practices in the future.

## Author Contributions

FH prepared the first draft of this editorial. All authors contributed to the article and approved the submitted version.

## Conflict of Interest

The authors declare that the research was conducted in the absence of any commercial or financial relationships that could be construed as a potential conflict of interest.
